# Clinical Prediction of Inadequate Vault in Eyes With Thick Lens After Implantable Collamer Lens Implantation Using Iris Morphology

**DOI:** 10.3389/fmed.2022.906433

**Published:** 2022-06-09

**Authors:** Zhikun Yang, Lihui Meng, Xinyu Zhao, Youxin Chen, Yan Luo

**Affiliations:** ^1^Department of Ophthalmology, Peking Union Medical College Hospital, Chinese Academy of Medical Sciences, Beijing, China; ^2^Key Lab of Ocular Fundus Diseases, Chinese Academy of Medical Sciences, Beijing, China

**Keywords:** iris, vault, ICL, abnormal shape, lens orientation

## Abstract

**Background:**

Obtaining an ideal vault is crucial in the implantable collamer lens (ICL) surgery. Prediction of the vault value is difficult since it requires the integration of multiple factors. The purpose of this study was to investigate the association between the iris shape and vault value in eyes with thick lens.

**Methods:**

The study was conducted in Peking Union Medical College Hospital. Patients who received ICL V4c between 2017 and 2021 were screened. Eyes with thick lens (>4.0 mm) and abnormal iris shape (concave or convex) were included. The preoperative biometric parameters and postoperative vault value were compared between eyes with concave shape group and convex shape group. The relationship between various factors and vault was assessed by spearman rank analysis and multiple linear regression analysis. Representative cases our strategies to deal with the abnormal vault were demonstrated.

**Results:**

Twenty eight eyes of 14 patients with thick lens and concave or convex shape iris were eventually included, with 14 eyes of 7 patients in group 1 (concave shape iris) and the other 14 eyes of 7 patients in group 2 (convex shape iris). The mean vault of group 1 was (0.16 ± 0.07) mm, which was significantly lower than (0.88 ± 0.13) mm in group 2. Multiple linear regression analysis showed iris shape (*P* < 0.001) was only the explanatory variables associated with the postoperative vault. In group1, 4 eyes showed extremely large ACA, requiring a secondary surgical intervention. So all of them underwent ICL exchange for a larger ICL. In group2, the ICL was implanted in a vertical or oblique position to avoid or rescue an extremely large vault.

**Conclusion:**

Concave shape iris had a higher risk of low vault and convex shape iris were more likely to demonstrate high vault in eyes with thick lens. Exchanging ICL for the larger size and adjusting ICL to the vertical or oblique orientation are good option to rescue the low or high vault, respectively.

## Introduction

The implantable collamer lens (Visian ICL; STAAR Surgical) is a type of phakic intraocular lens (IOL) used for correction of myopia and myopic astigmatism, which has been conducted in clinical practice for approximately two decades (1). A safer ICL with a small central hole (V4c, KS-AquaPORT, STAAR Surgical AG) has recently been developed (2). It makes iridectomies or iridotomies unnecessary and allows adequate aqueous flow to maintain the normal physiology of the anterior segment, which significantly decreases the incidence of anterior subcapsular opacities (3, 4).

Obtaining an ideal vault, the distance between the center of the posterior artificial lens surface and the center of the anterior crystalline lens surface, is crucial to ensure safety after ICL implantation. A low vault (<250 μm) could increase the risk of cataract formation, and a high vault (> 750 μm) may increase the risk of angle closure, pupillary block or pigment dispersion glaucoma (5). A proper ICL size is key to maintaining a safe vault and achieving a successful ICL implantation procedure. The calculation for ICL sizing mainly accords to horizontal corneal diameter [white-to-white (WTW)] and anterior chamber depth (ACD) values measurements. Other detailed anatomic dimensional parameters, including angel-to-angle (ATA), anterior chamber area or ciliary sulcus diameter [sulcus to sulcus (STS)], crystalline lens rise (LR) and thickness (LT) are also important to optimize the method of ICL sizing and improve the accuracy and precision of vault prediction (6–9). One of interesting findings is that the LT had a negative correlation with the vault value (10, 11). However, not all eyes with thick lens obtained a low vault since the prediction of vault required the integration of multiple factors.

Recently, with the wide application of ultrasound biomicroscopy (UBM) and anterior-segment optical coherence tomography (AS-OCT) in clinical practice, the inadequate vault which results from unmeasurable posterior chamber anatomic factors such as the ciliary body have been reported (12). Another important anatomic factor is related to iris. In the era before ICL V4c, the implanted lens had direct contact with the posterior surface of iris in 100% of the cases (13, 14). And the compression by the iris had large impacts on postoperative vault (15). Though the contemporary ICL V4c model resolved this situation, the influence of iris on vault value should not be ignored. It has been noticed that the iris could push the ICL down and warp it during miosis, to the extent that it adapted to the posterior surface of the iris, thus decreasing the central vault. And it is associated with LR (16). This indicates that iris morphology might influence the vault value to some degree. Therefore, on the basis of this phenomenon and our own experience, we hypothesized that morphology of iris might be associated with vault value. We categorized the iris shape into three groups, concave shape, convex shape and normal shape (17). For concave shape iris, it might cause low vault value. While for convex shape iris, it might cause high vault value.

Therefore, we designed and conducted this study to test the hypothesis mentioned above. To the best of our knowledge, this is the first study to report the possible relationship between the iris morphology and vault value and try to explain the mechanisms behind it.

## Materials and Methods

### Design

This retrospective study was performed in Peking Union Medical College Hospital and approved by the institutional review board. Patients who received ICL V4c surgery between 2017 and 2021 were screened. The study adhered to the tenets of the Declaration of Helsinki. All participants signed written informed consents before being enrolled in this study.

### Patient Data

Inclusion criteria were as follows: (1) age between 18 and 45 years; (2) spectacle spherical power, −2.50 to −20.00 D; (3) cylindrical power < 5.00 D; (4) stable refractive error ≥ 1 year; (5) corneal endothelial cell count ≥ 2,000 cells/mm^2^; (6) clear crystalline lens; (7) open angle on gonioscopy; (8) lens thickness > 4.0 mm. The exclusion criteria included any history of ocular pathologies, trauma, previous ocular surgeries, or chronic systemic diseases. All data were collected and evaluated by two retinal specialists (Zhikun Yang and Lihui Meng). For measurement data, they together took measurements three times and the average value was used for evaluation. For categorical data and descriptive data, evaluation was made separately and the disagreement was resolved by consulting the corresponding author (Yan Luo).

### Measurements

All patients underwent a complete ophthalmic examination, including uncorrected distance visual acuity (UDVA), corrected distance visual acuity (VA), manifest and cycloplegic refraction, non-contact tonometry, slit-lamp microscopy, endothelial cell density measurement, gonioscopy and funduscopic examination. The axial length (AL), LT and WTW were recorded from IOL Master 700. The angle-to-angle (ATA) distance, central corneal thickness (CCT), anterior chamber angle (ACA), and ACD were determined *via* AS-OCT. The STS distance was measured using UBM. UBM examinations were performed using an eyecup filled with stilled water after topical oxybuprocaine hydrochloride. The participants were required to lie down in a supine position and fixate on a ceiling target. A full view scan of the anterior segment was obtained at the 3–9 and 6–12 o’clock positions with the probe held perpendicular to the eyes. Radial scans of the limbus area through a typical process in the 2-, 4-, 8-, and 10- o’clock quadrants were also acquired. The iris shape was determined based on the UBM images. The vault value was acquired at least 1 month after the operation using AS-OCT. All patients received the examination before surgery and 1 day, 1 week, and 1 month, respectively, after the operation.

### Surgical Procedure

All surgeries were performed by a single experienced surgeon. A 3-mm clear corneal incision and a side hole were made after topical anesthesia. Then the anterior chamber was filled with a viscoelastic material. After that, V4c ICL was inserted through the corneal incision using an injector cartridge (STAAR Surgical AG) and placed in the posterior chamber. Finally, the viscoelastic material was completely removed and replaced with a balanced salt solution.

### Selection Process

**FIGURE 1:**
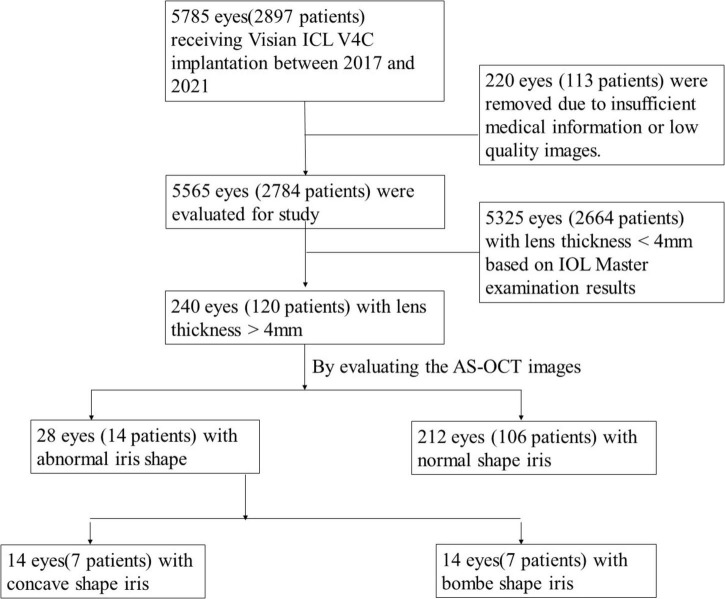
Study profile.

**FIGURE 2:**
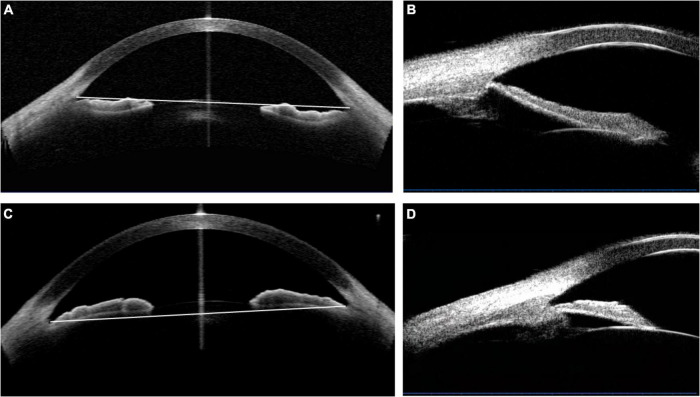
The determination of the concave and convex iris shape based on the anterior-segment optical coherence tomography (AS-OCT) or ultrasound biomicroscopy (UBM) images. **(A,B)** Concave shape iris: most part of iris locating behind angle-to-angle (ATA) with a concave shape of the iris pigment epithelium, referring to a “bowing” away from the cornea. And a wide sulcus could be detected in **(B)**. **(C,D)** Convex shape iris: most part of iris locating before ATA with a convex shape of the iris pigment epithelium, referring to that the mid-peripheral iris pigment epithelium is “bowed” toward the cornea. Besides, convex shape iris with anteriorly positioned ciliary body was demonstrated in **(D)**.

The selection process was shown in Figure 1. The concave iris was defined as a concave shape of the iris pigment epithelium, referring to a “bowing” away from the cornea; while the convex iris was characterized by a convex shape of the iris pigment epithelium, referring to that the mid-peripheral iris pigment epithelium is “bowed” toward the cornea (17) (Figure 2).

### Statistical Analysis

Statistical analyses were conducted with SPSS software version 25.0 (SPSS, Inc.). The VA was changed to logarithm of the minimum angle of resolution (logMAR) for statistical analyses. The continuous variables with a normal distribution were compared using the independent *t*-test, and data with a non-normal distribution were analyzed using the Mann-Whitney *U*-test. For categorical variables, the chi-square test was used. The relationship between the vault values and biometric parameters was evaluated using spearman rank analysis and multiple linear regression analysis. A *P*-value of less than 0.05 was considered statistically significant.

## Results

### General Data

**FIGURE 3:**
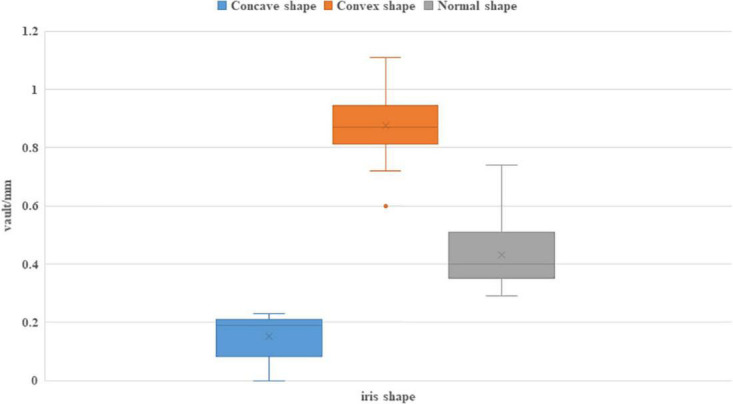
The vault distribution after ICL implantation of eyes with different iris shape.

Among patients who underwent ICL implantation surgery in our hospital during the analysis period, 5,785 eyes of 2,897 patients with detailed medical records were reviewed. 28 eyes of 14 patients with thick lens and concave or convex shape iris were included in this study. Besides, we randomly selected 8 patients (16 eyes) who had thick lens but with normal iris shape as the control group. Demographic and clinical baseline characteristics of these patients were described in Table 1. Eyes with concave shape iris (group 1), convex shape iris (group 2), and normal shape iris (group 3) were described separately. The postoperative assessment results which were obtained 1 month after the operation were documented. The vault distribution of all included eyes were shown in Figure 3.

**TABLE 1:** Demographic and clinical baseline characteristics in patients with concave shape iris and convex shape iris.

Parameter	Concave shape (group 1)	Convex shape (group 2)	Control group (group 3)	*P*-value (group 1 vs. 2 vs. 3)	*P*-value (group 1 vs. 2)	*P*-value (group 1 vs. 3)	*P*-value (group 2 vs. 3)
No. of eyes (patients)	14 (7)	14 (7)	24 (12)	/	/	/	/
Age (y)	28.29 ± 2.644 (25∼31)	31.57 ± 3.275 (27∼36)	36.75 ± 2.49 (32∼39)	<0.001*	*0.02* *	<0.001*	<0.001*
Sex (Female,%)	5/7 (71.4%)	4/7 (57.1%)	4/8 (75%)	0.741	1	1	0.608
UDVA (logMAR)	1.28 ± 0.26 (0.82∼1.70)	1.32 ± 0.35 (0.70∼2.00)	1.38 ± 1.56 (1∼2)	0.240	0.755	0.120	0.240
RE (D)	−9.482 ± 2.213 (−13.25∼−5.50)	−10.268 ± 4.713 (−19.5∼-4.25)	−10.28 ± 3.51 (−17.5∼−5)	0.853	0.579	0.552	0.822
IOP (mmHg)	13.00 ± 2.60 (9∼17)	15.14 ± 2.88 (11∼20)	16.36 ± 2.95 (11.5∼20.4)	*0.017* *	*0.049* *	*0.004* *	0.294
Keratometry	44.86 ± 2.35 (41.54∼48.21)	43.92 ± 1.36 (42.51∼46.77)	43.95 ± 1.14 (41.99∼45.64)	0.538	0.312	0.400	0.790
AL	26.63 ± 0.93 (25.22∼28.13)	27.28 ± 2.22 (24.46∼32.01)	27.25 ± 1.39 (24.83∼29.87)	0.355	0.662	0.131	0.473
ACD	3.05 ± 0.21 (2.85∼3.42)	2.90 ± 0.16 (2.57∼3.11)	3.13 ± 0.21 (2.91∼3.61)	*0.010* *	0.16	0.101	*0.002* *
ATA	11.80 ± 0.55 (10.86∼12.4)	11.69 ± 0.31 (11.09∼12.01)	11.82 ± 0.38 (11.11∼12.51)	0.424	0.251	0.667	0.313
WTW	11.70 ± 0.55 (10.7∼12.2)	11.53 ± 0.25 (11.2∼11.9)	11.81 ± 0.38 (11.3∼12.5)	*0.004* *	0.151	0.064	*0.001* *
ACA180°	59.35 ± 5.66 (52.5∼70.3)	37.21 ± 4.17 (29.8∼45.3)	50.64 ± 5.21 (42.1∼61.4)	<0.001*	<0.001*	<0.001*	<0.001*
ACA0°	59.71 ± 3.56 (55.3∼65.5)	38.76 ± 3.06 (32.7∼44.1)	52.84 ± 6.58 (40.5∼63)	<0.001*	<0.001*	*0.002* *	<0.001*
ACA (average)	59.53 ± 3.53 (55.85∼66.85)	37.99 ± 2.43 (34.5∼41.9)	51.74 ± 5.73 (41.3∼62.2)	<0.001*	<0.001*	<0.001*	<0.001*
LT	4.19 ± 0.13 (4.03∼4.41)	4.31 ± 0.17 (4.04∼4.62)	4.16 ± 0.12 (4.01∼4.38)	*0.027* *	0.051	0.608	*0.008* *
LR	0.24 ± 0.11 (0.11∼0.46)	0.31 ± 0.12 (0.13∼0.5)	0.14 ± 0.14 (−0.12∼0.39)	*0.003* *	0.106	*0.038* *	*0.001* *
Vault (mean ± SD)	0.16 ± 0.07 (0∼0.23)	0.88 ± 0.13 (0.6∼1.11)	0.43 ± 0.12 (0.29∼0.74)	<0.001*	<0.001*	<0.001*	<0.001*
Final UDVA	0.0025 ± 0.049 (−0.08∼0.10)	0.0216 ± 0.15 (−0.08∼0.40)	−0.04 ± 0.79 (−0.18∼0.10)	0.139	0.40	0.077	0.355
Final RE	0.0893 ± 0.252 (−0.25∼0.5)	0.14 ± 0.68 (−1.5∼0.75)	0.08 ± 0.27 (−0.5∼0.5)	0.126	0.09	0.951	0.085

ACA, anterior chamber angle; ACD, anterior chamber depth; AL, axial length; ATA, angle-to-angle; IOP, intraocular pressure; logMAR, logarithm of the minimum angle of resolution; LT, lens thickness; LR, lens rise; RE, refractive errors; UDVA, uncorrected distance visual acuity; WTW, white-to-white. Bold and italic values indicate the 0.001*.

In general, the mean age of these 22 patients was 32.41 ± 4.54 years old (ranging from 25 to 36). Female gender accounted for 59.1% (13/22). 14 eyes of 7 patients belonged to group 1, while the other 14 eyes of 7 patients belonged to group 2. The mean ages of three groups were 28.29 ± 2.64, 31.57 ± 3.28, and 36.75 ± 2.49, respectively. Patients in group 1 were significantly younger among these groups (*P* < 0.001). All three groups comprised females dominantly (71.4, 57.1, and 75%, respectively). As for ophthalmic examination results, there were no significant differences among three groups in the aspects of UDVA, refractive errors (RE), keratometry, AL, ATA, and LR. There were significant differences about intraocular pressure (IOP), ACD, WTW, and LT among three groups. Group 1 had significantly lower IOP compared with the other 2 groups. While group 2 had significantly larger ACD, smaller WTW, and higher LT. There were significant differences in ACA180°, ACA0°, and ACA (average) among three groups. The order lists of the mean value for these three parameters are the same: group1 > group 3 > group 2. The mean vault of group 1, 2, and 3 were (0.16 ± 0.07), (0.88 ± 0.13), and (0.43 ± 0.12) mm, respectively, which showed significant differences (*P* < 0.001).

### The Association Between Preoperative Biometric Parameters and Postoperative Vault

In our study, we defined that the vault value greater than 0.75 mm as the high vault group; the vault value less than 0.25 mm as the low vault group; otherwise they belonged to the normal vault group. We found that all eyes (14/14) in concave group acquired low vault after the first intervention; while 12/14 eyes in convex group acquired high vault. All eyes in normal shape group obtained normal vault. The spearman rank correlation analysis was conducted to evaluate the association between preoperative biometric parameters and exact vault value after ICL implantation, as is shown in Table 2. All variables whose correlation coefficient < −0.3 or > 0.3 in spearman rank correlation analysis were included in the multiple linear regression model. Four variables including WTW, ACA180°, ACA0°, ACA (average), and concave/convex iris shape were finally selected. Multiple linear regression analysis showed iris shape (*P* < 0.001) was only the explanatory variables associated with the postoperative vault.

**TABLE 2:** Results of spearman correlation analysis and multilinear regression analysis evaluating the association between preoperative biometric parameters and central vaulting after ICL implantation.

	Spearman rank analysis		Multilinear regression analysis		
Parameter	Correlation coefficient	*P*-value	Unstandardized coefficient	Standardized coefficient	*P-*value
Age	0.150	0.336			
Sex	0.154	0.324			
RE	0.028	0.858			
logMAR	−0.051	0.743			
IOP	0.152	0.330			
Keratometry	−0.134	0.392			
AL	0.037	0.816			
ACD	−0.210	0.177			
ATA	−0.179	0.250			
WTW	−0.312	0.042	−0.046	−0.067	0.334
ACA180°	−0.839	<0.001	−0.001	−0.034	0.802
ACA0°	−0.792	<0.001	−0.002	−0.061	0.666
ACA average	−0.838	<0.001			
LT	0.206	0.184			
LR	0.270	0.080			
Concave shape	−0.796	<0.001	−0.260	−0.392	<0.001
Convex shape	0.804	<0.001	0.379	0.581	<0.001

*ACA, anterior chamber angle; ACD, anterior chamber depth; AL, axial length; ATA, angle-to-angle; IOP, intraocular pressure; logMAR, logarithm of the minimum angle of resolution; LT, lens thickness; LR, lens rise; RE, refractive errors; UDVA, uncorrected distance visual acuity; WTW, white-to-white.*

### Implantable Collamer Lens Position and Exchanging

In group1, 4 eyes showed extremely large ACA, requiring a secondary surgical intervention. All of them underwent ICL exchange for a larger ICL. After the ICL exchange, they obtained an increased mean delta vault value (0.22 ± 0.11 mm).

In group2, the ICL was implanted in a vertical or oblique position to avoid an extremely large vault. Three eyes were operated in a horizontal position. However, the vault was quite high and the ACA was relatively small so that the second intervention was conducted to change the ICL position into the vertical direction. The decreased delta vault value of these three eyes was 0.20 ± 0.08 mm. Finally, 9 eyes were implanted in a vertical position and 5 eyes were implanted in an oblique position. The mean final UDVA and RE of these eyes receiving the second surgery were 0.03 ± 0.16 (−0.08∼0.40) and −0.043 ± 0.77 (−1.5∼0.75), respectively.

### Safety and Efficacy

The final UDVA and RE did not have significant differences among these three groups. For all patients in our study, the BCVA remained ideal and stable during the long period of follow-up (14–60 months). Besides, no complications such as elevated IOP, iris atrophy, pigment dispersion syndrome or cataract formation have been complained.

**FIGURE 4:**
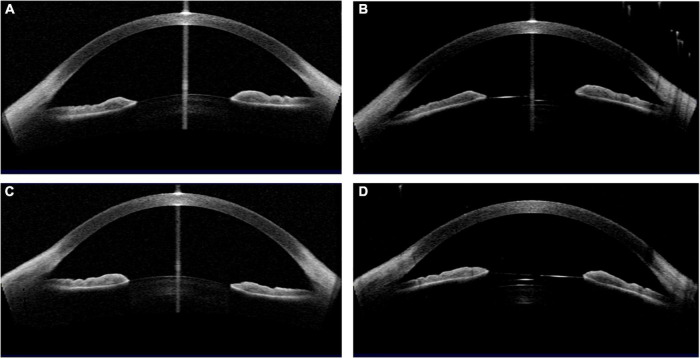
Case 1: **(A,C)** Concave shape iris before surgery; **(B,D)** low vault was shown in both eyes 1 year postoperatively. (Row 1: right eye; Row 2: left eye).

**FIGURE 5:**
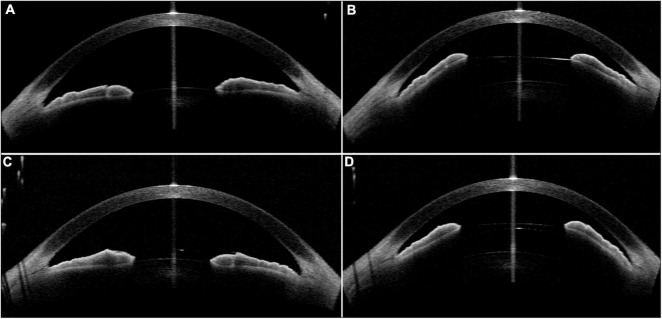
Case 2: **(A,C)** Convex shape iris before surgery; **(B,D)** high vault was shown in both eyes 1 month after ICL implantation. (Row 1: right eye; Row 2: left eye).

**FIGURE 6:**
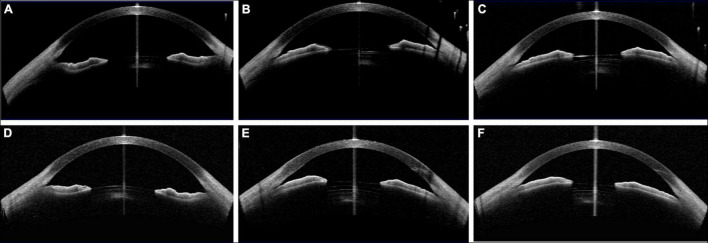
Case 3: **(A,D)** Concave shape iris before surgery; **(B)** low vault was shown 1 day postoperatively with 121 ICL; **(C)** the vault was slightly increased after ICL exchange into 126 ICL 1 month postoperatively; **(E)** the vault of the left eye was larger than that of the right eye with 126 ICL; **(F)** the vault decreased 1 month postoperatively. (Row 1: right eye; Row 2: left eye).

**FIGURE 7:**
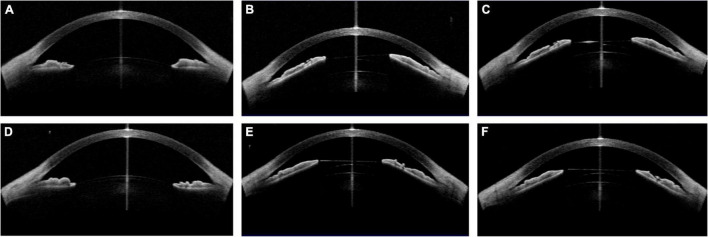
Case 4: **(A,D)** Convex shape iris before surgery; **(B,E)** 1 month after ICL implantation (B oblique position, E vertical position); **(C)** the vault value decreased after changing the ICL into a vertical position; **(F)** 2 month after ICL implantation. (Row 1: right eye; Row 2: left eye).

The representative cases were shown in Table 3 and Figures 4–7.

**TABLE 3:** The clinical characteristics and biometric parameters of 4 representative cases.

	Age	Sex	laterality	RE	BCVA (logMAR)	ACD	CCT	WTW	ATA	AL	LT	Surgery design	BCVA (logMAR)	Vault (mm)	Second intervention	Follow-up time (months)	Final vault (mm)
case 1	25	F	OD	−13.25DS−2.00DC*20	0.10	3.29	545	12.2	12.4	28.13	4.41	137 ICL (oblique 40°)	0.10	0.17	None	54	0.08
			OS	12.5DS−2.25DC*165	0.10	3.34	552	12.2	12.4	27.97	4.37	137 ICL (horizontal)	0.10	0.19	None	54	0.11
case 2	27	F	OD	−5.25DS−1.0DC*165	0	2.97	565	11.5	11.84	25.74	4.31	132 ICL (vertical)	0	1.11	None	24	0.96
			OS	−5.75DS−0.75DC*170	0	2.93	563	11.5	11.71	25.87	4.25	132 ICL (vertical)	0	0.94	None	24	0.94
case 3	31	F	OD	−8.5DS−0.50DC*180	0	2.95	534	10.7	10.94	25.62	4.39	121 ICL (horizontal)	0	0.09	ICL exchanging into 126ICL	15	0.25
			OS	−8.5DS	0	2.89	530	10.7	10.86	25.22	4.19	121 ICL (horizontal)	0	0	ICL exchanging into 126ICL	15	0.23
case 4	36	M	OD	−19.5DS−1.25DC	0.40	2.6	505	11.8	11.82	32.01	4.23	132 ICL (oblique 45°)	0.40	0.82	Adjust into the 90°	14	0.51
			OS	−19.5DS−1.5DC	0.52	2.57	502	11.8	12	31.89	4.62	132 ICL (vertical)	0.52	0.6	None	14	0.54

*ACA, anterior chamber angle; ACD, anterior chamber depth; AL, axial length; ATA, angle-to-angle; BCVA, best corrected visual acuity; DS, spherical **equivalent; DC,** cylinder **equivalent;** IOP, intraocular pressure; logMAR, logarithm of the minimum angle of resolution; LT, lens thickness; LR, lens rise; RE, refractive errors; UDVA, uncorrected distance visual acuity; WTW, white-to-white.*

## Discussion

Though ICL implantation is an overall safe and effective option for surgical correction of high myopia, postoperative complications are reported, most of which were associated with the inappropriate vault (5, 18). Therefore, obtaining an ideal vault is of vital importance to ensure the safety and efficacy after ICL implantation. Many preoperative biometric factors are considered important to predict postoperative vault. However, the accurate prediction of postoperative vault still remains a challenge. In this study, we focused on those eyes with thick lens receiving ICL implantation, exploring the influence of abnormal shape iris on postoperative vault and proposing our strategies to solve the problems of inadequate vault.

Our results demonstrated a significant association between iris shape and postoperative vault in eyes with thick lens. For concave shape iris, it had a tendency to demonstrate low vault value after ICL implantation; while for convex shape iris, most of eyes acquired a high vault value. With regards to the parameters about the lens, previous studies have reported that the LT and LR had significantly negative correlation with vault value in previous studies (10, 11, 19, 20). However, eyes in our study obtained both high and low vault value even though they all had the LT > 4.0 mm. This indicated that other important factors influenced the vault value greatly. Herein, the iris shape demonstrated significant correlation with the vault value. As is known, the ICL size had a positive association with postoperative vault (11). Therefore, it seemed rational to choose a relative larger size ICL when facing the increased LT or LR.

This phenomenon that iris shape influenced the vault value might be explained from the following aspects. Firstly, it might be partially caused by the relationship between iris and ciliary body. Chen et al. investigated the factors related to ciliary body morphology in clinical prediction of excessive vault. They found that the iris-ciliary angle had a negative correlation with postoperative vault. Each degree reduction of iris-ciliary angle was significantly associated with 4% increased odds of vault greater than 1,000 μm. Noteworthy, if the eyes had smaller iris-ciliary angle, the iris was more likely to demonstrate a convex shape morphology. On the contrary, in eyes with larger iris-ciliary angle or wide ciliary sulcus, the support from ciliary body for iris might be compromised, which led to its concave shape (12). Secondly, the postoperative location of the ICL haptics also contribute to the vault inappropriateness. Several researchers have investigated the exact positions of ICL haptics. García-Feijoó et al. reported that most ICL haptics were finally located in the ciliary sulcus or ciliary body (21). Choi et al. found 64.7% or ICL haptics were inserted in the ciliary sulcus (22). In Zhang et al.’s study using full-scale UBM, they found that there were various positions of ICL in the posterior chamber and the haptics were inserted at different positions, which had a significant influence on postoperative vaulting. If the incorrectly placed haptics in the ciliary process caused abnormal structure of ciliary sulcus, the ICL might shift downwards and acquired a low vault value. While the eyes with haptics on the top of the ciliary sulcus were more likely to have a high vault value. The different positions of ICL haptics was probably due to the ICL size choice preoperatively and invisible intraoperatively (23). The operator could not see the back of the iris directly during the operation, leading to the haptics wrong-placed, which induced the ICL abnormal arching. We assumed that the abnormal iris shape might probably increase the risk of abnormal position of haptics, resulting in abnormal structure of ciliary sulcus more likely. Therefore, clinical prediction of vault value just based on the UBM examination results could ignore some factors intraoperatively or postoperatively. A comprehensive analysis is required when predict the vault value.

In addition, this study proposed our strategies to deal with the inadequate vault and found a reasonable solution. For low vault value, exchanging operation with a larger size ICL is a good option. In our study, 4 eyes obtained the low vault value after ICL implantation and demonstrated extremely large ACA, which required exchanging surgery for a larger ICL. After the second intervention, all these eyes obtained an ideal vault and no decreased visual acuity or other complications were reported during the follow up time. The method that ICL exchanging to a larger size ICL for low vault was supported by the positive correlation between ICL size and vault value (11, 24). As for high vault after ICL implantation, we made a rotation of the ICL from horizontal to oblique or vertical orientation and obtained an ideal vault eventually. This method was demonstrated to be effective in several studies (18, 25, 26). The reason was that the sulcus has a vertically oval shape, with the vertical diameter longer than the horizontal one (27, 28).

Before the era of ICL V4c, the horizontal compression of the ciliary sulcus was thought to be a key factor in vault formation, but it could not effectively predict vault when the iris produced the vertical compression. Vertical compression would push the ICL toward the crystalline lens and the ICL haptics toward the ciliary sulcus, leading to a buffering effect and subsequent a less than expected vault (15). Combining with our results, we presumed that the abnormal iris shape might disturb the flow of the aqueous humor or influence the haptics position, inducing an invisible force of vertical compression. Thus, adjust the ICL to a vertical or oblique orientation could be considered as an option to avoid the high vault value.

There are several limitations in this study. Firstly, this study was conducted with a small sample size in a single center, which precluded the results to be generalized. Secondly, not all patients received UBM examination, which might cause inadequate evaluation of the ciliary body and influence the decision of surgery design. Thirdly, patients visited the clinics at different time points after surgery, thus some statistical analyses related to the postoperative parameters could not be performed. Finally, eyes with LT more than 4.0 mm composed a small proportion of all subjects, restricting the results to be popularized.

## Conclusion

In conclusion, we described the association between abnormal iris shape and postoperative vault in eyes with thick lens. Concave shape iris presented a higher risk of low vault and convex shape iris were more likely to demonstrate high vault. Exchanging ICL for the larger size and adjusting ICL to the vertical or oblique orientation are good option to rescue the low or high vault value, respectively. These findings offer clinicians a new insight about the vault prediction and ICL sizing. Further studies with large sample size and prospective design are needed.

## Data Availability Statement

The raw data supporting the conclusions of this article will be made available by the authors, without undue reservation.

## Ethics Statement

The studies involving human participants were reviewed and approved by the institutional review board of Peking Union Medical College Hospital. The patients/participants provided their written informed consent to participate in this study.

## Author Contributions

ZY carried out the entire procedure including the collection of medical records, image evaluation, and manuscript drafting. LM contributed in image evaluation, statistical analysis, and manuscript drafting. XZ and YC helped draft the manuscript. YL conceived of the study, coordinated and participated in the entire process of drafting, and revising the manuscript. All authors read and approved the final manuscript.

## Conflict of Interest

The authors declare that the research was conducted in the absence of any commercial or financial relationships that could be construed as a potential conflict of interest.

## Publisher’s Note

All claims expressed in this article are solely those of the authors and do not necessarily represent those of their affiliated organizations, or those of the publisher, the editors and the reviewers. Any product that may be evaluated in this article, or claim that may be made by its manufacturer, is not guaranteed or endorsed by the publisher.
